# Incidence and Characteristics of Patients with Visual Impairment due to Macular Edema Secondary to Retinal Vein Occlusion in a Representative Canadian Cohort

**DOI:** 10.1155/2012/723169

**Published:** 2012-10-14

**Authors:** Robert J. Petrella, Julie Blouin, Brian Davies, Martin Barbeau

**Affiliations:** ^1^Aging, Rehabilitation and Geriatric Care Program, Lawson Health Research Institute, London, ON, Canada N6C 2R5; ^2^Individual Health Outcomes Inc., ON, Canada; ^3^Parkwood Hospital, Aging, Rehabilitation and Geriatric Care Research Center, B-3002, 801 Commissioners Rd E., London, ON, Canada N6C 5J1; ^4^Outcomes Research, Novartis Pharmaceuticals Canada Inc., Dorval, QC, Canada H9S 1A9

## Abstract

Retinal vein occlusion (RVO) is an obstruction of the retinal venous system, and macular edema (ME) is a complication of RVO that can lead to blindness. The Canadian incidence of visual impairment (VI) due to ME secondary to RVO is unknown. This observational, retrospective study used records from the Southwestern Ontario database to observe the annual incidence, demographics, and comorbidity characteristics of patients with VI due to ME secondary to RVO. From 47,166 patients, 73 with RVO (>40 years old) were identified: 53 with branch retinal vein occlusion (BRVO), 20 with central retinal vein occlusion (CRVO). The annual incidence of VI (visual acuity <20/40 in Snellen equivalent) due to ME secondary to BRVO was (mean (95%CI)) 0.056% (0.011–0.072), and to CRVO was 0.021% (0.008–0.081). Furthermore, a greater proportion of RVO patients had hypertension (68% versus 14%) or dyslipidemia (16% versus 10%), when compared to a healthy control cohort of 76,077 subjects (*P* < 0.05). This study presents a description of the characteristics of patients with VI due to ME secondary to RVO in a real-world Canadian setting. The results demonstrate that BRVO was more frequent than CRVO, and that RVO in this patient population was associated with several vascular comorbidities.

## 1. Introduction

Retinal vein occlusion (RVO) is an obstruction of the retinal venous system, it has an abrupt onset and is an important cause of visual morbidity [1]. Retinal vein occlusions constitute the second most common cause of retinal vascular disease after diabetic retinopathy, with a prevalence of between 1% and 2% in persons older than 40 years of age [2–5]. In the Wisconsin Beaver Dam Eye Study, 12% of eyes that developed severe visual impairment (best corrected visual acuity ≤20/200) during a 15-year followup were due to RVO [6]. Although the exact etiology of RVO is not known, it is likely to follow a thrombotic event, possibly caused by external compression or disease of the vein wall [1, 7]. 

RVO can be categorized as branch retinal vein occlusion (BRVO), if the obstruction is located in one of the branches of the central vein; or central retinal vein occlusion (CRVO), if it is located in the central vein, at the level of the optic nerve [8]. BRVO encompasses a heterogeneous group of disorders with different clinical aspects [9] and presents with dilated and tortuous retinal venous system in a particular quadrant or hemisphere of the retina and is often associated with macular edema [10]. CRVO is presented with hemorrhagic changes in all four quadrants of the retina and dilated and tortuous veins [10]. In both BRVO and CRVO, cotton wool spots, disc edema, and neovascularization may also be present [10]. 

The incidence of RVO has been estimated to be 0.12%/year in adults aged ≥45 years for BRVO and 0.04%/year in adults aged ≥45 years for CRVO in Caucasian populations [6, 11]. However, the Canadian incidence of visual impairment (VI) due to macular edema (ME) secondary to RVO is unknown. This study aims to determine the annual incidence of VI and characteristics of patients with ME secondary to BRVO and CRVO in a real-world Canadian setting.

## 2. Methods

An observational, retrospective study was conducted. Records from a longitudinal population-based database of more than 170,000 patients in 53 family practice clinics in Southwestern Ontario, Canada were analyzed between January 1, 2008 and December 31, 2009. These records contained chart-abstracted information such as visit diagnosis, medications, and consultation notes. 

In order to compare characteristics and comorbidities of patients with diabetic macular edema (DME) to those of the general population, a control cohort was constructed by matching age and gender for all patients in the database >18 years by clinic location.

Initial extractions of control cohort and RVO patients with ME, (defined as retinal thickening within 500 *μ*m of the macular center) and VI (defined as best corrected visual acuity ≤20/40 in the RVO eye), were accomplished utilizing International Classification of Disease codes (ICD9/ICD10), reviewing patient charts for text entries of symptoms that supported a diagnosis of RVO and concomitant comorbidity, and reviewing patient treatment records unique to RVO including consultation notes and hospital discharge summaries. Demographic characteristics and comorbidities were reported. Data included in this study comprised patient characteristics and demographics, cardiovascular comorbidity and events, and medication coverage. 

### 2.1. Description and Validity of the Southwestern Ontario (SWO) Database

The SWO database has recorded patient level data on the clinical diagnoses at each visit, symptoms corroborating the diagnoses, clinical data, prescribed treatments including lifestyle interventions and medications, physician visits, hospitalizations, and diagnostic/laboratory test results, allowing for the conduct of patient level analyses, since 2000. Data from the 53 practices participating in the SWO database cohort are routinely updated on a quarterly basis with immediate reconciliation at the point of care. The quarterly activity is triggered by chart entry and billed activity for patient encounters. All practices included in the SWO database are part of a family practice research network involved in various audit and clinical research activities. Practices have consented to centralized accrual of clinical data from the patient record (UWO IRB 09572). All records are anonymous and conform to current confidentiality industry standards. Each patient's Ontario Health Insurance Plan (OHIP) number is assigned a unique patient identification number in the SWO database. To protect the privacy of patient's medical information, a 128-bit SSL certificate is installed on the production SWO Web server. The industry standard data protection method ensures the security of data during transmission across the Internet.

Validation studies of the SWO database confirming the quality and completeness of the recorded data show good agreement between estimates of the prevalence of cardiovascular risk factors obtained from the SWO database and other published estimates [12–15]. Moreover, there is a correlation between the SWO database and national data (i.e., IMS, Brogan PharmaStat) on the utilization of prescription medication (personal communication, Petrella; Kamino, IMS). 

### 2.2. Statistical Analysis

Starting from the index date (the date of the first diagnosis of RVO during the study period until December 2009), all subjects meeting inclusion/exclusion criteria were analyzed to understand the demographics and treatment patterns of care. For treated patients, the treatment choices were characterized, and the treatment pattern was related to clinical characteristics of patients, including type of drug coverage (public, private, out of pocket). 

For continuous variables, the mean, standard deviation, median, minimum and maximum values were estimated. For categorical variables, the number and percentage of each category within an assessment was calculated for non-missing data. A 95% confidence interval is provided for proportions. Visual impairment (VI) is defined as visual acuity <20/40. 73 of 47,166 patients over 40 years of age with new diagnosis of RVO and a control cohort of 76,077 patients were extracted for this analysis.

## 3. Results

### 3.1. Demographics

Please refer to Table 1 for data on demographics for the control and RVO cohorts. The average age of those with new occurrences of RVO was 61 years. Examination of recorded episodes during the observation period by gender and age revealed the following: only 8% of patients with a new diagnosis of RVO during this time were males between the ages of 40–59 years, compared with 43% of females in that age group. Ninety-two percent of males with new diagnoses of RVO were over the age of 60, compared to 54% of females. A higher percentage of Caucasian (81%) and Aboriginal (11%) ethnic groups were affected by RVO compared to their respective groupings (78% and 9%) within the control cohort.

Please refer to Table 2 for data on disease characteristics for the control and RVO cohorts. More RVO patients were overweight (23%) or obese (13%) compared to the control cohort. More RVO patients were smokers (18%) compared to control. More RVO patients had hypertension (68% versus 14%) or dyslipidemia (16% versus 10%) than control cohort (*P* < 0.05). One quarter of RVO patients had a history of vascular disease, primarily MI and stroke. Fifteen percent of patients with RVO suffer from chronic kidney disease. 

Please refer to Table 3 for data on incidence and type of RVO within the RVO cohort. Seventy-three patients over 40 years old with a new diagnosis of RVO were identified from 47,166 patients over 40 years old. Fifty-three patients had BRVO, and 20 patients had CRVO as interpreted from consultation notes. The annual incidence of VI due to ME secondary to BRVO and CRVO was found to be 0.056%, 95% CI (0.011–0.072) and 0.021%, 95% CI (0.008–0.081), respectively.

As shown in Table 4, majority of patients had public drug coverage.

## 4. Discussion

Macular edema (ME) is a complication of RVO that can lead to blindness. This was the first study to assess the incidence of VI due to ME secondary to BRVO and CRVO and to describe the disease characteristics of patients with RVO in a Canadian setting. 

 A few other studies have assessed the incidence of RVO. In the Australian Blue Mountains Eye Study, the 5-year incidence of any RVO was 1.0%, and the 10-year incidence was 1.6% [11]; in the US, the 5-year incidence was 0.8%, and the 15-year incidence was 2.3% [6]; and in Japan, the 9-year incidence was reported at 2.0% [16]. The incidence of BRVO appears generally higher than the incidence of CRVO. In all these studies, as in ours, the small number of individuals that developed incident RVO makes it difficult to know with accuracy the ratio of incident BRVO to incident CRVO and to use these estimates as a basis for comparison [1]. More males (92%) over 60 years versus 54% females had the diagnosis of RVO. These findings are consistent with findings in other studies—RVO is rarely seen in individuals younger than 50, but may affect up to 5% of individuals over the age of 80—as noted by Laouri et al. in a review of the literature on the burden of retinal vein occlusion [1]. 

Consistent with previous literature, we found that patients with RVO were more likely to present hypertension, dyslipidemia, or vascular diseases compared with general population [4, 17–19]. Further, our findings concur with previous studies that found risk factors more common in patients with BRVO [8]. 

As observed by Lattanzio et al., given the close association of RVO with systemic vascular disease, new patients with RVO should be evaluated for hypertension, diabetes, and lipid abnormalities, as it may be “the presentation of significant vascular morbidity” [8]. In the case of younger patients, who may be otherwise healthy, the pathogenesis and risk factors are still poorly understood and additional evaluation of coagulation disorders and a history of thromboses may be necessary [8, 20]. 

This study has some limitations. This retrospective study was conducted utilizing ICD9/ICD10 disease codes and reviewing patient charges and treatment records, including consultation notes and hospital discharge summaries. BRVO and CRVO are easily detected using standard ophthalmological diagnostic tools and techniques [21], so while the diagnosis and classification of RVO are valid, difficulties in interpreting data contained in consultation notes, as well as data concerning number of episodes requiring consultation and treatment during the observation period, may have resulted in some inconsistencies in data capture.

## 5. Conclusions

This paper presents a description of the characteristics of patients with VI due to ME secondary to BRVO and CRVO in a real-world Canadian setting. Consistent with findings in other studies, RVO in this patient population was associated with several vascular comorbidities. BRVO is more frequent than CRVO. The annual incidence of VI due to ME secondary to BRVO and CRVO was estimated to be 0.056% and 0.021%, respectively. 

## Figures and Tables

**Table 1:** RVO in patients across age, sex, and ethnic origin.

	Control cohort (*N* = 76,077)	Patients with RVO (*N* = 73 out of 47,166 persons >40 years)
Sex [*n* (%)]		
Male	37551 (49%)	35 (48%)
Female	38526 (51%)	38 (52%)
Average age at diagnosis (years)	n/a	57
Average age (years)	61	61
Age distribution [*n* (%)]		
Males <40	14455 (19%)	0%
Males 40–59	12933 (17%)	6 (8%)
Males 60–69	3083 (5%)	30 (41%)
Males 70+	6086 (8%)	37 (51%)
Females <40	14454 (19%)	2 (3%)
Females 40–59	11411 (15%)	31 (43%)
Females 60–69	3804 (5%)	23 (32%)
Females 70+	9129 (12%)	16 (22%)
Ethnicity [*n* (%)]		
Caucasian	59340 (78%)	58 (81%)
Aboriginal	6847 (9%)	8 (11%)
Hispanic	3043 (4%)	1 (2%)
South Asian	3055 (5%)	3 (4%)
Asian	1521 (2%)	1 (1%)
African descent	1506 (2%)	0 (0%)

**Table 2:** Disease characteristics of patients with RVO.

	Control cohort (*N* = 76,077)	Patients with RVO (*N* = 73 out of 47,166 persons)
Average age at diagnosis (years)	n/a	57
Overweight (BMI = 25–29.9 kg/m^2^) [*n* (%)]	3799 (5%)	17 (23%)
Obesity (BMI ≥ 30 kg/m^2^) [*n* (%)]	1522 (2%)	9 (13%)
BMI, male (mean ± SD)	25 ± 13.9	28 ± 10
BMI, female (mean ± SD)	24 ± 14.1	29 ± 9
Family history, type 2 diabetes [*n* (%)]	782 (1%)	2 (3%)
Smoking [*n* (%)]	3804 (5%)	13 (18%)
Hypertension [*n* (%)]	10650 (14%)	49 (68%)
Systolic blood pressure (mean ± SD)	131 ± 15.4	143 ± 15
Diastolic blood pressure (mean ± SD)	77 ± 8.5	87 ± 10
Dyslipidemia [*n* (%)]	7603 (10%)	11 (16%)
History of impaired fasting glucose (IFG) [*n* (%)]	0 (0%)	0 (0%)
History of impaired glucose tolerance (IGT) [*n* (%)]	780 (1%)	1 (1%)
Glycosylated haemoglobin (HbA1C) (mean ± SD)	6.3 ± 0.3	7.6 ± 1.3
History of vascular Disease [*n* (%)]	9129 (12%)	18 (25%)
Acute coronary syndrome (ACS) [*n* (%)]	4567 (6%)	5 (7%)
Myocardial infarction (MI) [*n* (%)]	799 (1%)	9 (12%)
Stroke [*n* (%)]	714 (1%)	4 (6%)
Percutaneous coronary transluminal arthroplasty (PCTA) [*n* (%)]	689 (1%)	1 (1%)
Coronary artery bypass graft (CABG) [*n* (%)]	701 (1%)	3 (3%)
Peripheral arterial disease (PAD) [*n* (%)]	215 (<1%)	2 (2%)
Congestive heart failure (CHF) [*n* (%)]	755 (1%)	0 (0%)
History gestational diabetes (females) [*n* (%)]	181 (<1%)	0 (0%)
Chronic kidney disease [*n* (%)]		
Stage 1	0 (0%)	3 (3%)
Stage 2	0 (0%)	3 (3%)
Stage 3	737 (1%)	2 (3%)
Stage 4	2282 (3%)	4 (5%)
Stage 5 (end-stage renal disease)	766 (1%)	1 (1%)
Dialysis	(<1%)	0 (0%)

Microvascular complications (%)	5%	0%

**Table 3:** RVO type.

Patients with RVO (new diagnoses) (*N* = 73/47,166)		
Type (*n*)	BRVO 53	CRVO 20
Annual incidence	.056%	.021%
	(0.011%–0.072%)	(0.008%–0.081%)

**Table 4:** Payer type.

Payer type	Control cohort (*N* = 76,077)	Patients with RVO (*N* = 73/47,166)
Public [*n* (%)]	72273 (95%)	64 (88%)
Private [*n* (%)]	9890 (13%)	9 (12%)
Out of pocket [*n* (%)]	350 (<1%)	1 (1%)
